# Patterns of statin adherence in primary cardiovascular disease prevention during the pandemic

**DOI:** 10.3389/fphar.2022.980391

**Published:** 2022-11-14

**Authors:** Sara Malo, Lina Maldonado, María José Rabanaque, Antonio Gimeno-Miguel, Sara Castel-Feced, María Jesús Lallana, Isabel Aguilar-Palacio

**Affiliations:** ^1^ Department of Preventive Medicine and Public Health, University of Zaragoza, Zaragoza, Spain; ^2^ Grupo de Investigación en Servicios Sanitarios de Aragón (GRISSA), Fundación Instituto de Investigación Sanitaria de Aragón (IIS Aragón), Zaragoza, Spain; ^3^ Network for Research on Chronicity, Primary Care, Health Promotion (RICAPPS), ISCIII, Madrid, Spain; ^4^ Department of Applied Economics, University of Zaragoza, Zaragoza, Spain; ^5^ EpiChron Research Group, Aragon Health Sciences Institute (IACS), IIS Aragón, Miguel Servet University Hospital, Zaragoza, Spain; ^6^ Primary Care Pharmacy Service, Sector Zaragoza III, Servicio Aragonés de Salud (SALUD), Zaragoza, Spain

**Keywords:** medication adherence, statin, chronic disease, healthcare system, disease management, computer modeling, cluster analysis, COVID-19

## Abstract

**Background:** Study of medication adherence patterns can help identify patients who would benefit from effective interventions to improve adherence.

**Objectives:** To identify and compare groups of statin users based on their adherence patterns before and during the COVID-19 pandemic, to characterize the profile of users in each group, and to analyze predictors of distinct adherence patterns.

**Methods:** Participants of the CARhES (CArdiovascular Risk factors for HEalth Services research) cohort, comprising individuals aged >16 years, residing in Aragón (Spain), with hypertension, diabetes mellitus and/or dyslipidemia, took part in this observational longitudinal study. Individuals who began statin therapy during January–June 2019 were selected and followed up until June 2021. Those with a cardiovascular event before or during follow-up were excluded. Data were obtained from healthcare system data sources. Statin treatment adherence during the implementation phase was estimated bimonthly using the Continuous Medication Availability (CMA9) function in the AdhereR package. Group-based trajectory models were developed to group statin users according to their adherence pattern during July 2019–June 2021. Group characteristics were compared and predictors of each adherence pattern were analyzed using multinomial logistic regression.

**Results:** Of 15,332 new statin users, 30.8% had a mean CMA9 ≥80% for the entire study period. Four distinct adherence patterns were identified: high adherence (37.2% of the study population); poor adherence (35.6%); occasional use (14.9%); and gradual decline (12.3%). The latter two groups included users who showed a change in adherence (increase or decrease) during the pandemic emergence. Users with suboptimal adherence were likely to be younger, not pensioners, not institutionalized, with low morbidity burden and a low number of comorbidities. Female sex and switching between statins of different intensity increased the likelihood of belonging to the occasional use group, in which improved adherence coincided with the pandemic.

**Conclusion:** We identified four distinct adherence patterns in a population of new statin users; two of them modified their adherence during the pandemic. Characterization of these groups could enable more effective distribution of resources in future similar crisis and the routine implementation of patient-centered interventions to improve medication adherence.

## 1 Introduction

In line with current recommendations (Visseren et al., 2021) statins are widely prescribed for prevention of cardiovascular disease (CVD). However, while statin efficacy in primary prevention of CVD has been well demonstrated in clinical trials, their effectiveness in clinical practice is less clear. This is in part because the desired clinical effects are only achievable if the patient adheres to the treatment plan (Chaure-Pardos et al., 2022). Adherence to long-term therapies for chronic illnesses has been described as suboptimal (Menditto et al., 2018), particularly in the case of statins for primary CVD prevention (Ofori-Asenso et al., 2018).

In addition to poor health outcomes, nonadherence is associated with increased healthcare costs and reduced patient quality of life (Hassan et al., 2021). A recent study of a cohort of statin users showed that, after adjusting for patient characteristics, poor adherence increased the probability of preventable healthcare utilization and spending, especially among minorities and groups with low socioeconomic status (Zhang et al., 2022). Conversely, noncontinuous access to both healthcare services and medications may jeopardize adherence and self-care behavior and, consequently, effective management of chronic conditions (Ágh et al., 2021).

The last 2 decades have seen a growing emphasis placed on the lack of transparency in the operationalization of medication adherence measures, and on the overabundance of terms used to describe medication use (Arnet et al., 2016). This complicates comparison of adherence findings across studies and their translation to real-world clinical practice. In 2012, to overcome potential confusion and misunderstanding, the European-funded Ascertaining Barriers to Compliance (ABC) project proposed a new medication adherence taxonomy (Vrijens et al., 2012). The ABC taxonomy, which has been widely adopted internationally, subdivides adherence into three essential elements: initiation, implementation, and discontinuation. Thus, poor medication adherence can occur in the following situations or combinations thereof: non-initiation of the prescribed treatment after its prescription; suboptimal implementation of the dosing regimen; and discontinuation of treatment (nonpersistence). In the study of implementation (i.e., the degree to which the patient’s dose corresponds to the prescribed dose regimen), application of group-based trajectory modeling (GBTM) is increasingly used, as it constitutes a powerful tool with which to represent adherence behaviors using longitudinal data (Librero et al., 2016; Walsh et al., 2021). Given the dynamic nature of adherence patterns, which can vary over time, the superiority of this approach over classical adherence point estimators, expressed as mean values, is evident. Indeed, certain circumstances can induce changes in the adherence patterns of patients with relatively constant behaviors.

The COVID-19 pandemic has impacted the management and behavior of chronic patients due to changes in lifestyle (diet, physical activity, alcohol and tobacco consumption) and social situation (stress, anxiety, social isolation). Similarly, changes in the organization and provision of healthcare resources have likely influenced the continuity of care received by these patients (Palmer et al., 2020; Lau and McAlister, 2021). Given that the aforementioned parameters are all considered determinants of medication adherence (Kardas et al., 2013), analysis at a population level of the implementation adherence during the different stages of the pandemic could help identify the most affected groups of patients. This information in turn could be used to facilitate better distribution of resources in the context of future crises, helping avoid such negative impacts on patient medication adherence.

The objectives of this study were 1) to compare adherence patterns before and during the COVID-19 pandemic among adults in Aragón, Spain, taking statins for primary CVD prevention, 2) to describe the individual, clinical, and therapeutic characteristics of users in each group and 3) to analyze predictors of distinct adherence patterns.

## 2 Materials and methods

### 2.1 Study design and setting

This observational longitudinal study was conducted among participants of the CARhES (CArdiovascular Risk factors for HEalth Services research) cohort. This is a population-based dynamic cohort of individuals aged >16 years, registered as users of the Aragón Health System, with hypertension, diabetes mellitus and/or dyslipidemia. Information collected from this cohort includes quantitative real-world data extracted from administrative databases from the healthcare system.

Aragón is an Autonomous Community located in the northeast of Spain with a population of 1.3 million inhabitants. It has a high level of aging, with more than 20% of the population aged >64 years (Instituto Aragonés de Estadística. Gobierno de Aragón, 2022). In Spain, the health system is based in the principles of universal, equitable, free access and fairness of financing, and is predominantly funded by taxes (Bernal-Delgado et al., 2018). The 17 Spanish Autonomous Communities, to which healthcare competences have been devolved, manage most of the public health resources. Primary care constitutes the core element of the health system, and encompasses the majority of health care, health maintenance, health recovery, rehabilitation, and social work activities. Pharmaceutical care, one of the services provided by the National Health Service, covers all medicines and health products that are approved, registered, and eligible for reimbursement, and ensures that patients receive the correct formulation and dose of their medication at the lowest possible cost (Bernal-Delgado et al., 2018). Management of medication adherence is overseen by doctors (prescribers); primary care nurses (who supervise adherence and side-effects); pharmacists (who dispense medications and supervise treatment adherence and early detection of side-effects). However, routine assessment of adherence is not mandatory in the management of chronic patients, nor are specific adherence support programs widely offered on a routine basis.

### 2.2 Study population and data sources

In the present study, participants in the CARhES cohort identified as new statin users during the period January–June 2019 were followed-up until June 2021. New statin users were defined as those who had not received any statin prescription during the 6-month period preceding the date of treatment initiation. Analyses were restricted to participants treated exclusively with statins (Anatomical Therapeutic Chemical [ATC] codes C10AA [plain statins], C10BA [statins in combination] and C10BX [statins in combination with other drugs]), and not with other lipid-lowering agents in monotherapy, during the period January 2019 to June 2021. From those selected, we excluded individuals with a diagnosis of a major adverse cardiovascular event before or during the study period, as defined by a diagnosis of acute myocardial infarction, nontraumatic intracranial hemorrhage, or cerebral infarction (codes I21, I22 and I60–I63; International Classification of Diseases, 10th Revision) during hospitalization. Individuals who died during follow-up were also excluded.

Data were obtained from BIGAN, a platform for the secondary use of health data from the Aragón Health System. BIGAN provides pseudonymized individual level patient’s data from the following information systems: Users Database, which records sociodemographic information including age, sex, pharmacy copayment level, type of pharmaceutical provision, type of economic activity, and institutionalization status; Pharmaceutical Dispensation Database, which records the dispensing date, ATC code, number of pills per package and the number of packages dispensed by pharmacies and covered by the Aragón Health System; Minimum Basic Data Set database, which records diagnoses and dates of hospitalizations; Emergency Database, which gathers diagnoses and dates of visits to emergency services; Primary Care Database, which records information on visits to primary care and corresponding medical diagnoses; Adjusted Morbidity Groups, which records diagnostic data collected from the Minimum Basic Data Set and the Primary and Emergency Care Databases, including the total number of chronic diseases and affected systems, the morbidity burden (obtained through aggregation of all the patient’s diagnoses), and the presence of specific chronic morbidities such as hypertension, diabetes, and depression. This information is later reviewed, cleansed and integrated to feed the CARhES cohort.

Socioeconomic level was determined based on pharmacy copayment level and type of economic activity. Based on the combination of these two variables, seven mutually exclusive categories were created: employed individuals earning <€18,000 per annum (p.a.); employed individuals earning ≥€18,000 p.a.; individuals receiving unemployment allowance; individuals with a contributory pension <€18,000 p.a.; individuals with a contributory pension ≥€18,000 p.a.; individuals receiving free medicines (those with minimum integration income or who no longer receive unemployment allowance); and other situations not included in the aforementioned categories.

Based on the first statin prescribed during the follow-up period, individuals were classified as “high-intensity statin users” (i.e., those receiving atorvastatin or rosuvastatin and combinations thereof) or “low–moderate intensity statin users” (i.e., those receiving simvastatin, lovastatin, pravastatin, fluvastatin or pitavastatin and combinations thereof). Based on this, we created a new variable which identified users who switched from low–moderate to high intensity statin use and *vice versa*. In cases in which more than one switch occurred during the study period, only the first was considered.

### 2.3 Estimation of adherence

Statin implementation adherence was assessed in the study population from July 2019 to June 2021 using two different approaches: first, as a summary estimation of adherence, calculated using AdhereR, a package in the R-free software environment developed for transparent and reproducible analysis of electronic healthcare data (Dima and Dediu, 2017); and second, as a dynamic longitudinal measure that allows grouping of statin users based on their adherence pattern or trajectory.

The conceptualization of adherence was performed according to the consensus-based Medication Adherence Reporting Guideline (EMERGE) (de Geest et al., 2018) and the TEOS framework (Dima et al., 2021). The latter was developed as a guide to the conceptual analysis of adherence Timelines and key Events in relation to research Objectives and data Sources in order to improve the transparency and reproducibility of adherence studies.

#### 2.3.1 Measurement of summary adherence

Adherence was estimated bimonthly in statin users. AdhereR implements a set of functions that are consistent with current adherence guidelines, definitions, and operationalizations. It allows the computation of nine different versions of the Continuous measure of Medication Availability (CMA), a summary adherence estimate which can be mapped onto Medication Possession Ratio (MPR) and Proportion of Days Covered (PDC), with the advantage of allowing the selection of different analysis options according to health conditions and types of medication.

In this study, the CMA9 function was computed as the number of days of theoretical medication use divided by the duration of the adherence assessment period, allowing for carryover of supply from before and during this period and excluding the supply left at the end. CMA9 differs from other CMA indicators in that it assumes persistence, based on which it adjusts implementation. CMA9 computes a ratio of days’ supply for each individual in the study period, and then weighs all days by their corresponding ratio to generate a mean adherence value that remains constant from one supply until the next or until the end of the assessment period (Dima and Dediu, 2017; Allemann et al., 2019). CMA9 was computed for repeated sliding windows within the adherence assessment period. These sliding windows had a duration of 2 months (the usual period between dispensations in the study region), without overlaps.

Given that the usual prescribed statin dose is one pill per day, the number of days of medication supplied was estimated based on the number of pills contained in the package(s) dispensed (i.e., 28 or 30, depending on the statin). During a hospitalization period, it was assumed that treatment was supplied by the hospital, and therefore the remaining supplies were extended accordingly.

The mean adherence (CMA9 value) was calculated in the study population. Also, the mean CMA9 indicator obtained for each statin user was dichotomized using an arbitrary cut-off of 0.8 (i.e., 80%).

#### 2.3.2 Adherence trajectory groups

The bimonthly CMA9 estimates were incorporated into GBTM, which grouped patients based on their adherence patterns. For this purpose, longitudinal data were clustered by performing K-means analysis (Allemann et al., 2019). The optimal number of groupings was selected based on the Calinski & Harabasz criterion, considering the Genolini variant (Genolini et al., 2015). This is a non-parametric criterion that can be calculated without any previous hypothesis on data.

### 2.4 Statistical analysis

Sociodemographic, clinical, and treatment characteristics of the study population were described using the mean and standard deviation (SD) or median and interquartile range (IQR) for continuous variables, and frequency and percentage for categorical variables. The frequency and percentage of users with a mean composite CMA9 ≥80% was estimated.

To achieve the first objective of grouping statin users according to their adherence pattern from July 2019 to June 2021, GBTM was conducted. Next, key pandemic dates were identified and linked with the evolution of adherence patterns.

In order to achieve the second objective, the same individual, clinical, and treatment characteristics described above were compared between statin users within each trajectory group. Continuous covariates, depending on their parametric distribution, were compared using either a Student’s *t*-test or analysis of variance (ANOVA), and categorical variables using the Chi-squared or Fisher’s exact test.

Finally, multinomial logistic regression was performed to identify the sociodemographic, clinical, and treatment factors associated with belonging to each group, answering the third objective.

## 3 Results

The characteristics of the study population are described in the 3.1 subsection. The following subsections (3.2, 3.3 and 3.4) respond, respectively, to the three main study objectives.

### 3.1 Patient characteristics

Data from 15,332 individuals were analyzed. All were new statin users with neither prior cardiovascular events nor cardiovascular events or death during the follow-up period. Mean age was of 60.6 (SD, 13.2) years. Table 1 presents additional sociodemographic and clinical data.

**TABLE 1:** Characteristics of the study population.

Characteristics	N = 15,332
Sex, n (%)	
Women	7,903 (51.5%)
Age, n (%)	
16–44 years	1,670 (10.9%)
45 to 64 years	7,901 (51.5%)
65 to 79 years	4,369 (28.5%)
≥80 years	1,392 (9.1%)
Socioeconomic level, n (%)	
Employed, < €18,000 p.a	2,457 (16.0%)
Employed ≥ €18,000 p.a	3,172 (20.7%)
Pensioner < €18,000 p.a	2,567 (16.7%)
Pensioner ≥ €18,000 p.a	2,894 (18.9%)
Unemployed	695 (4.5%)
Free medicines	2,736 (17.8%)
Other	811 (5.3%)
Institutionalized, n (%)	243 (1.6%)
Number of chronic diseases, mean (SD)	4.1 (2.3)
Number of affected systems, mean (SD)	3.1 (1.5)
Morbidity burden, mean (SD)	6.8 (4.2)
Comorbidities, n (%)	
Hypertension	6,443 (42.7%)
Diabetes	2,932 (19.4%)
Depression	2,401 (15.9%)
Statin switching during the study period, n (%)	
High to low–moderate intensity statins	245 (1.6%)
Low–moderate to high intensity statins	728 (4.7%)
No switching	14,359 (93.7%)
Adherence (CMA9), mean (SD)	0.5 (0.4)
Mean adherence (CMA9) ≥ 0.8, n (%)	4,724 (30.8%)

Abbreviations: N, number; p.a., per annum; SD, standard deviation.

The mean morbidity burden was estimated in individuals for whom information was available (total, 15,088).

A total of 4,724 (30.8%) new statin users showed a mean adherence (CMA9) of at least 0.8 (80%). Of the total study population, 6.3% switched from low–moderate to high intensity statins or *vice versa* during the study period (Table 1).

### 3.2 Adherence trajectories

**FIGURE 1:**
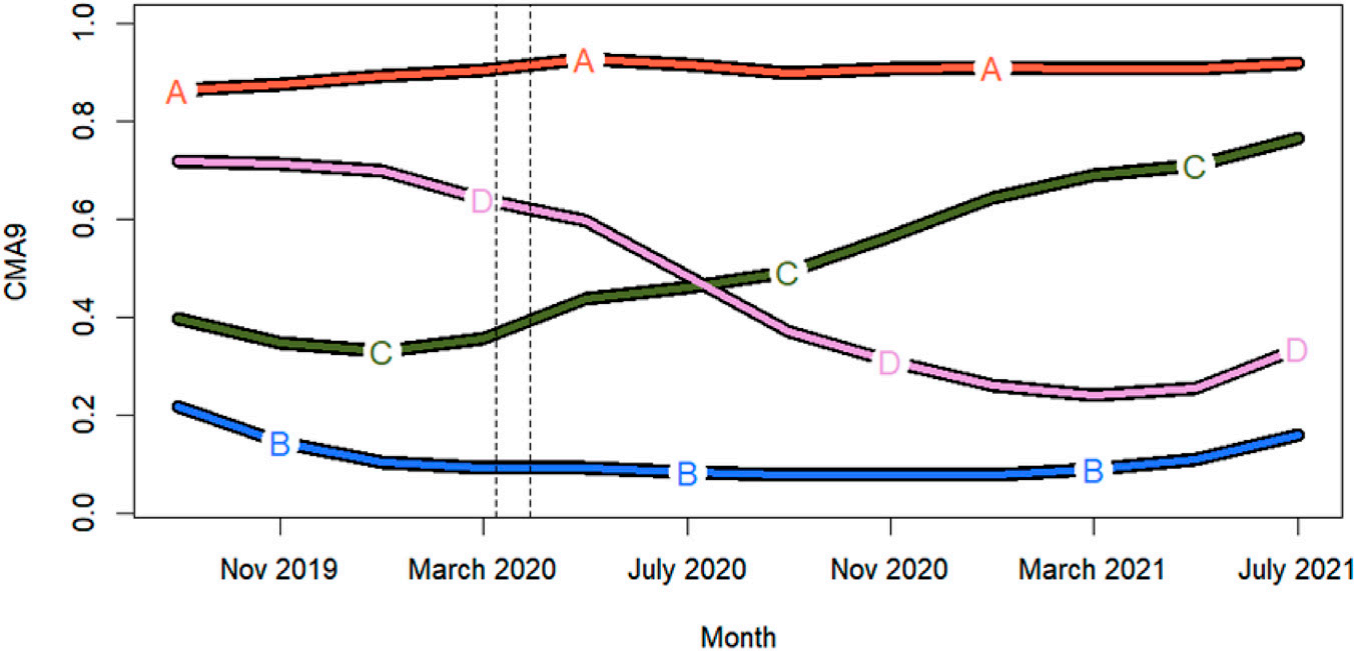
Patient groups according to adherence trajectory: A (37.2% of study population); B (35.6%); C (14.9%); and D (12.3%). Dashed lines indicate the strict COVID-19 lockdown implemented in Spain from March 15 to April 26 2020.

The method used estimated the optimal number of clusters as 4. Thus, the following adherence trajectories were identified within the study population (Figure 1):

Group A: High and constant adherence.

Group B:Poor adherence, without significant variations.

Group C: Occasional use, with a trend towards improved adherence from March 2020.

Group D: Gradual decline, with a sharp decrease between March 2020 and March 2021.

In two groups of statin users (C and D) a change in the adherence pattern coincided with the onset of the COVID-19 pandemic (specifically, the strict lockdown implemented in Spain).

### 3.3 Characterization of the identified groups

Table 2 presents the sociodemographic, clinical and treatment characteristics, including adherence, of the four groups of statin users. Comparison of most of the characteristics across user groups revealed statistically significant differences. In general, statin users in the high adherence group (group A) were older, with a higher proportion of pensioners and institutionalized individuals, a higher mean number of chronic pathologies and affected systems, and a higher morbidity burden. Hypertension and diabetes were also more frequent in this group. Conversely, statin users in the poor adherence group (group B) were more likely to be aged 16–44 years, employed receiving <€18,000 p.a., with fewer comorbidities and a lower morbidity burden. Individuals in the occasional users and the gradual decline trajectories (groups C and D, respectively) presented intermediate characteristics in terms of age, socioeconomic level, and comorbidity profile. One remarkable finding was the higher proportions of women and of users who switched statin treatment (especially those who switched from a low–moderate to a high intensity statin [10.1%]) in group C.

**TABLE 2:** Comparison of characteristics of statin users in each group.

Characteristics	Group A (n = 5,702)	Group B (n = 5,460)	Group C (n = 2,284)	Group D (n = 1,886)	*p*-value
Sex, n (%)					
Women	2,931 (51.4%)	2,713 (49.7%)	1,271 (55.6%)	988 (52.4%)	<0.001
Age, n (%)					
16–44 years	337 (5.9%)	872 (16.0%)	226 (9.9%)	235 (12.5%)	<0.001
45–64 years	2,897 (50.8%)	2,805 (51.4%)	1,214 (53.2%)	985 (52.2%)	
65–79 years	1941 (34.0%)	1,290 (23.6%)	633 (27.7%)	505 (26.8%)	
≥80 years	527 (9.2%)	493 (9.0%)	211 (9.2%)	161 (8.5%)	
Socioeconomic level, n (%)					<0.001
Employed < €18,000 p.a	920 (16.1%)	1,358 (24.9%)	464 (20.3%)	430 (22.8%)	
Employed ≥ €18,000 p.a	809 (14.2%)	944 (17.3%)	386 (16.9%)	318 (16.9%)	
Pensioner < €18,000 p.a	1,104 (19.4%)	805 (14.7%)	375 (16.4%)	283 (15.0%)	
Pensioner ≥ €18,000 p.a	1,305 (22.9%)	826 (15.1%)	419 (18.3%)	344 (18.2%)	
Unemployed	216 (3.8%)	292 (5.4%)	95 (4.2%)	92 (4.9%)	
Free medicines	1,116 (19.6%)	869 (15.9%)	422 (18.5%)	329 (17.4%)	
Other	232 (4.1%)	366 (6.7%)	123 (5.4%)	90 (4.8%)	
Institutionalized, n (%)	121 (2.1%)	61 (1.1%)	29 (1.3%)	32 (1.7%)	<0.001
Number of chronic pathologies, mean (SD)	4.3 (2.3)	3.9 (2.2)	4.1 (2.2)	4.0 (2.3)	<0.001
Number of affected systems, mean (SD)	3.3 (1.5)	3.0 (1.5)	3.1 (1.5)	3.1 (1.5)	<0.001
Morbidity burden, mean (SD)	7.3 (4.4)	6.4 (4.0)	6.8 (4.0)	6.7 (4.4)	<0.001
Comorbidities, n (%)					
Hypertension	2,749 (48.4%)	1986 (37.4%)	955 (42.7%)	753 (40.4%)	<0.001
Diabetes	1,305 (23.0%)	848 (16.0%)	429 (19.2%)	350 (18.8%)	<0.001
Depression	897 (15.8%)	802 (15.1%)	387 (17.3%)	315 (16.9%)	0.063
Switching during study period, n (%)					<0.001
High to low–moderate intensity statins	83 (1.5%)	47 (0.9%)	76 (3.3%)	39 (2.1%)	
Low–moderate to high intensity statins	238 (4.2%)	154 (2.8%)	231 (10.1%)	105 (5.6%)	
No switching	5,381 (94.4%)	5,259 (96.3%)	1977 (86.6%)	1742 (92.4%)	
Mean adherence (CMA9) ≥0.8, n (%)	4,724 (82.8%)	0 (0%)	0 (0%)	0 (0%)	0.000

SD, standard deviation; CMA, continuous medication availability; p.a., per annum.

The mean morbidity burden was estimated in individuals for which information was available (total, 15,088).

**FIGURE 2:**
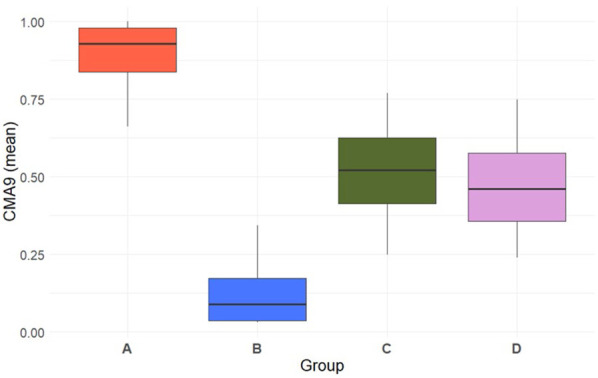
Boxplot depicting mean adherence (CMA9) for each statin user group.

Figure 2 presents the mean adherence for each group of statin users. Mean adherence differed between groups A (0.9) and B (0.1), but not between groups C and D (both with 0.5).

### 3.4 Predictors of the different adherence patterns

**FIGURE 3:**
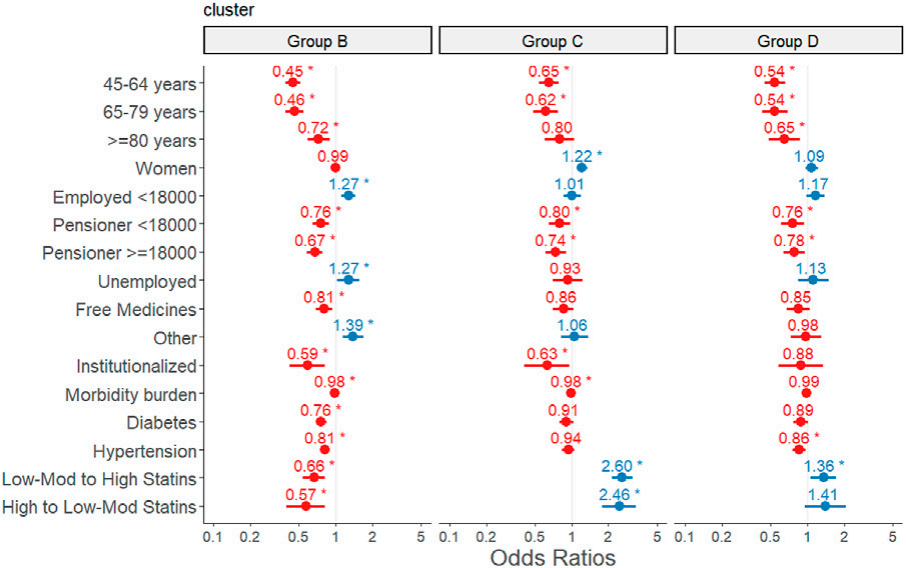
Predictors of inclusion in the poor adherence (group B), occasional use (group C) and gradual decline (group D) groups in relation to the high adherence group (group A). Multinomial logistic regression analysis. The final adjusted model included 15,088 individuals, for whom information on all the variables studied was available. The reference category in the dependent variable was the high adherence group (group A). For predictors, the reference categories were: 16–44 years (age), men (sex), employed earning ≥€18,000 per annum (socioeconomic level), not institutionalized, no diabetes, no hypertension, no switching from low–moderate to high intensity statins or *vice versa*.

Potential predictors of inclusion in a given adherence trajectory are shown in Figure 3.

Compared with statin users in the high adherence group (group A), those in the poor adherence (group B), occasional users (group C), and gradual decline (group D) groups were, in general, more likely to be young (16–44 years), neither pensioners nor free medicine recipients, not institutionalized, with a low morbidity burden and no comorbidities such as diabetes or hypertension. These associations were statistically significant in most cases (Figure 3). A significant association with sex was observed only for the occasional use group (group C), members of which were more likely to be women (OR 1.22, 95%CI 1.10–1.35) compared with the high adherence group (group A). Inclusion in the occasional use or gradual decline groups (C and D) was positively associated with switching from a low–moderate to a high intensity statin (OR 2.60, 95%CI 2.15–3.15 and OR 1.36, 95%CI 1.07–1.72, respectively). Inclusion in group C was also associated with switching from a high to a low–moderate intensity statin (OR 2.46, 95%CI 1.79–3.38). Conversely, patients in the poor adherence group (group B) were less likely to switch statin treatment than those in the high adherence group (group A).

## 4 Discussion

In this real-world data study, we assessed implementation of treatment in new statin users during the period 2019–2021 using software specially developed for reproducible analysis of electronic healthcare data. GBTM identified four distinct adherence trajectories in the study population before and during the COVID era. We analysed the characteristics most associated with nonadherent patterns as well as changes in adherence that occurred during critical phases of the pandemic. These findings can help further our knowledge of the effect of the pandemic on adherence to preventive treatment, which is one of the most important pillars in the management of CVD risk factors.

Our study population was made up of individuals with no previous cardiovascular events who started statin treatment during the first 6 months of 2019 in the Spanish region of Aragón. Participants were predominantly mostly middle-aged and older, with a moderate morbidity burden and a high rate of other CVD risk factors. Mean adherence was 50%, and 30.8% of participants had a mean adherence ≥80%. Previous studies have reported poor statin adherence (Yeaw et al., 2009; Menditto et al., 2018), as well as a high degree of variability in adherence rates among populations. Although 80% is the most common cut-off point for dichotomizing adherence, this is an arbitrary value that should be adapted to each disease and treatment. In any case, its application can be useful to estimate the proportion of new statin users with suboptimal adherence.

We identified four distinct statin adherence trajectories during the follow-up period from July 2019 to June 2021. To date, few studies have applied GBTM to classify users of a particular drug into different groups according to their adherence utilization pattern. Among the few studies that have used this approach, the number of distinct trajectories identified ranges from 3 to 4 (Librero et al., 2016; Hickson et al., 2020; Majd et al., 2021). These numbers depend on the sample size, the type of medication, and the characteristics of the study population. In a population-based cohort of patients discharged after hospitalization for coronary heart disease, Librero et al. applied GBTM to groups of users of statins, among other medications, based on their adherence trajectories over time. They identified three different adherence patterns for statins: adherent (74.9% of patients); occasional users (17.5%); and fast decline (7.6%). Compared with the present findings, the authors grouped a much higher proportion of statin users into the highly adherent trajectory (74.9% vs. 37.2%). However, our study population differed to that of Librero et al. in that our patients had not experienced a previous cardiovascular event. And taking statins for primary CVD prevention has been associated with increased nonadherence (Ofori-Asenso et al., 2018). In a population of new statin users already treated with antihypertensive drugs, Majd et al., 2021 analyzed the possible association between past medication-taking behavior and current statin adherence pattern. They found that previous trajectories of adherence to antihypertensive drugs predicted future statin adherence patterns, suggesting that the routine study of adherence during the first year of treatment initiation could provide valuable information to stakeholders to develop tailored interventions to improve adherence. In our particular case, statin users in groups B and D may benefit most from improvement strategies. Numerous interventions to improve adherence during the implementation phase have been carried out in different contexts, but suffer from methodological limitations in terms of design and have reported only modest effects on medication adherence (Cross et al., 2020; Yang et al., 2022). In Spain, such interventions are not routinely implemented in general practice.

Our multinomial regression analyses showed that being older, a pensioner, and having a higher morbidity burden were associated with high and constant adherence over time. Conversely, young users, employed earning <€18,000 p.a. or unemployed, with no comorbidities, were more likely to be included in the poor adherence trajectory. Factors related to mild symptoms have previously been associated with a poorer adherence profile (Kardas et al., 2013). Furthermore, those in the high adherence group more frequently had access to free medicines than those in the other groups. Requiring copayment has already been described as a predictor of nonadherence in other studies (Librero et al., 2016; Ofori-Asenso et al., 2018), as it represents a barrier to access to chronic treatments, especially in patients with a low socioeconomic status.

Comparison of the poor adherence (group B) with the high adherence (group A) trajectories showed that, in statin users with a constant non-adherent pattern, a switching in statin treatment did not lead to an increase in the adherence levels. One feasible explanation for this observation is a lack of concern among statin users in group B about their high cholesterol levels, given their asymptomatic condition. These users may also attempt to control their disease by means of other behaviors such as diet modification and physical activity. Finally, it is also possible that a lack of in-person consultations at the beginning of the COVID-19 pandemic may have caused patients to neglect their condition, with consequent negative health outcomes. In any case, further studies will be necessary to identify the underlying reasons, and to assess the validity of prescribing statins to low-risk patients who continuously show poor adherence, resulting in poor statin effectiveness (Chaure-Pardos et al., 2022). For them, alternative non-pharmacological measures might be a more appropriate choice.

Being a woman increased the likelihood of inclusion in the occasional users trajectory (group C) and, therefore, of improving statin adherence during the study period. Although our finding cannot be easily compared with previous studies, given repeated inconsistency in the association between sex and adherence pattern (Kardas et al., 2013), this association is nonetheless interesting. Group C consisted mainly of statin users with poor adherence in the months preceding the pandemic who subsequently improved their medication-taking behavior, almost reaching adherence values of 80% by the end of the follow-up period. Differently from the observed in statin users in group B, with permanent poor adherence, the higher frequency of switching between statins within users in group C could indicate an active patient–health professional relationship and also explain the positive effect on adherence. A more in-depth study of the characteristics and circumstances of these patients could help unravel the uneven impact of COVID-related changes on adherence patterns in different population groups.

The COVID-19 pandemic completely disrupted the healthcare of patients with chronic diseases, postponing face-to-face appointments or replacing them with telemedicine services. Ágh et al. (2021) found that in-person consultations were limited during the pandemic in 90% of 38 European countries studied. This limitation, together with social distancing restrictions imposed in Spain, may have negatively influenced continuous access to medication, which is a prerequisite for appropriate adherence. In Spain, electronic prescribing is widely available, and the prescribing of chronic therapies was automatically renewed even during the worst phases of the pandemic. However, even though face-to-face consultations were not essential for medication prescribing and supply, virtual or telephonic care suffer from several disadvantages compared with in-person consultation (e.g., they do not allow optimal involvement of the patient in shared decision-making, education, and self-management) (Lewis et al., 2016). In order to maintain treatment adherence during pandemic lockdown, some authors proposed measures such as home delivery of prescription medications for older, frail patients with a high-risk mental state, for whom leaving the house was particularly challenging. Longer-duration prescriptions that facilitate medication access, especially for patients living in remote areas, could also be prioritized (Ágh et al., 2021). Although these changes may be required in exceptional situations, such as the COVID-19 pandemic, they should always be balanced against the risk of not providing high-quality care. The creation of e-health systems to support patients in long-term treatment and the development and implementation of a patient-centered care model are possible solutions to avoid deterioration of self-care and medication adherence in similar situations in the future (Palmer et al., 2020). Indeed, with a view to improving the care of patients receiving chronic treatments, Spanish primary care professionals have routine access to information on patient prescription and dispensation records. This allows the healthcare professional to check the end date of the last prescription refill, which serves as a proxy of patient adherence, and to intervene if necessary. Coordination between health and social services has been acknowledged as one of the cornerstones of the management of chronic patients in risky situations, underscoring the importance of providing integrated patient-centered care (Rodriguez-Blazquez et al., 2020).

### 4.1 Strengths and limitations

The main strength of this study is its population-based nature. The analysis of real-world data from all new statin users in a population of this size lends the findings a high degree of validity. Another strength is the use of AdhereR*,* which has been developed to aid the computation of electronic healthcare data-based adherence estimates within the widely used open-source environment R, and to promote transparency and comparability of research findings. Moreover, this approach allows the application of the sliding window function to the CMA9 indicator to describe the use of medication during the implementation adherence phase. The consensus-based TEOS framework (Dima et al., 2021) suggests the estimation of individual-level patterns during this phase in a short-to-medium time frame if temporal within-patient variations affecting medication adherence are to be captured, as in the present study. GBTM offers certain advantages over traditional methods of adherence assessment, in which medication adherence is considered a static, rather than dynamic and longitudinal, process. GBTM also offers greater accuracy and validity in the design of adherence interventions, given that conventional methods provide irregular or variable patterns (e.g., those obtained in groups C and D) that would return a similar mean adherence measure for the entire study period if temporal adherence dynamics were not considered. Paradoxically, these groups with a more irregular pattern of use would likely benefit most from an improvement intervention. Furthermore, the identification of characteristics associated with poor or intermediate statin adherence patterns could facilitate strategies that are more focused on the necessary actions. For instance, patients with poor adherence from the beginning could benefit from negotiation with the prescriber when deciding upon treatment and dose, or from an explanation about the advantages and possible adverse effects associated with their medication. Conversely, in patients who start treatment with acceptable adherence that subsequently diminishes (the gradual decline group), further exploration of the underlying factors is required. The onset of the pandemic led to many changes at the levels of the individual, society at large, and healthcare systems, all of which may have contributed to decreased adherence. To investigate these contributions further, and thereby address the situation, it would be desirable to have additional information beyond the variables analyzed in the present study.

This study has several limitations. First, the use of electronic health databases is limited by the quality of the data recorded. However, the health data platform used in the present study has already been used in multiple studies conducted by different research groups. Our data source did not include certain variables that could have been of interest as potential predictors of nonadherence. Nonetheless, the available information allowed us to identify several important factors related to statin adherence and to broaden our knowledge of the issue. The assessment of statin adherence was performed based on data derived from pharmacy claims. Given that patients do not necessarily consume all the drugs purchased from the pharmacy, our approach may have overestimated the true consumption of statins. However, this limitation is common to all studies using these types of data sources. The use of the AdhereR package also presents some minor limitations: its creators have acknowledged certain aspects of the program that can be improved, and will likely be addressed in future versions (Dima and Dediu, 2017). The modeling process used in the present study involves several choices that may have influenced the final results (e.g., the option to carry-over into the observation window and the selection of sliding windows of 2 months in the GTBM). Finally, when interpreting findings, it should be noted that a reduction in adherence does not always imply inappropriate patient behavior, and may reflect a medical indication to stop treatment or even switch to another low-lipid lowering drug.

### 4.2 Future implications

Both the existing literature and the present findings indicate frequently poor adherence among patients treated with statins for CVD primary prevention. Furthermore, even statin users with an optimal adherence pattern can be affected by exceptional situations such as that resulting from the recent COVID-19 pandemic. Poor statin adherence could be explained by the fact that hyperlipidemia is a non-symptomatic process, for which patients do not have the urgent need for treatment, and by the frequent adverse effects of statins. This casts doubt on the appropriateness of prescribing statins as first-line treatment in certain circumstances or to patients with individual or clinical characteristics associated with a higher risk of nonadherence. For this reason, it is extremely important to continue furthering our knowledge of factors that may facilitate adherence, in particular during implementation of the prescribed regimen, given the suboptimal results of numerous interventions to improve adherence conducted in different contexts. Knowledge resulting from collaborative research initiatives focused on the topic, such as the European Network to Advance Best practices and technoLogy on medication adherencE (ENABLE), is particularly valuable for the application of practices related to medication adherence. Supporting funding of collaborative cross-country projects is therefore an important course of action.

The development, improvement and promotion of free tools such as AdhereR in adherence studies as well as the routine application of consensus-based scales, taxonomies, and guidelines to medication adherence studies will also ensure progress in standardizing adherence estimators and approaches and greater comparability of results obtained in different populations. This is one of the keys to improving the utilization of chronic therapies, using as a reference those healthcare system interventions that produce the best adherence-related outcomes.

During the decade preceding the pandemic, public health efforts focused on improving healthcare system coordination and providing guidance on the management of chronic conditions and lifestyle factors. The resilience of the healthcare system was one of its most acknowledged characteristics. However, after the unprecedent situation caused by the COVID-19 pandemic, structural reforms in the healthcare systems, including the Spanish system, may be required to prioritize actions to improve chronic care management, address the basic needs of patients with chronic diseases, and minimize the potentially devastating impact of the COVID-19 outbreak on especially vulnerable individuals. Proposed actions include: ensuring the continuity of healthcare services; increasing equitable access to educational materials (e.g., ehealth) that promote awareness and to local and social support activities; and facilitating monitoring by healthcare professionals (including telemedicine). Medication nonadherence is a multifactorial process, and therefore should be supervised and influenced by a range of healthcare professionals. Defining the roles and functions of each professional, as well as increasing public funding, are essential in order to carry out successful interventions to improve medication-taking behavior. In the particular case of statins, indication should always be dependent on the patient’s clinical situation. However, the risk of nonadherence, based on the patient’s characteristics, could be assessed to guide prescribing decision-making, and exhaustive adherence monitoring implemented, based on which adjustments can be made if necessary to help achieve optimal adherence.

## 5 Conclusion

The COVID-19 pandemic has changed everyday life, and has had a marked impact on individuals with chronic diseases whose management and self-care depend on multiple social, individual, and healthcare-related factors. In this large-scale pharmacoepidemiological study of statin users, we found that one-third of the study population did not take statins as prescribed during the 2-year follow-up period. This observation sheds doubt on the appropriateness of statin indication in individuals with this profile (i.e., young, healthy, and employed earning <€18,000 p.a.). For individuals fitting this profile, recommendation of non-pharmacological measures might be a more effective and efficient alternative. On the other hand, almost one-third of the study population changed their medication-taking behavior during the pandemic period, in some cases showing a decline in statin adherence. Characterization of statin users with a poor adherence pattern enables the effective design and implementation of interventions to enhance medication adherence using person-centered approaches and to distribute resources to avoid repeated negative effects on adherence in these patients in future crises.

## Data Availability

The data analyzed in this study is subject to the following licenses/restrictions: All data used in this study pertain to the CARhES cohort. While these data are not publicly available due to their sensitive nature, interested researchers can nonetheless contact the corresponding author to request access. Requests to access these datasets should be directed to smalo@unizar.es.
